# A Longitudinal Operant Assessment of Cognitive and Behavioural Changes in the *Hdh^Q111^* Mouse Model of Huntington’s Disease

**DOI:** 10.1371/journal.pone.0164072

**Published:** 2016-10-04

**Authors:** Emma Yhnell, Stephen B. Dunnett, Simon P. Brooks

**Affiliations:** 1 Neuroscience and Mental Health Research Institute, Cardiff University 3rd Floor, Hadyn Ellis Building, Maindy Road, Cardiff, CF24 4HQ, United Kingdom; 2 The Brain Repair Group, Cardiff University School of Biosciences, The Sir Martin Evans Building, Museum Avenue, Cardiff, CF10 3AX, United Kingdom; Queen Mary University of London, UNITED KINGDOM

## Abstract

Huntington’s disease (HD) is characterised by motor symptoms which are often preceded by cognitive and behavioural changes, that can significantly contribute to disease burden for people living with HD. Numerous knock-in mouse models of HD are currently available for scientific research. However, before their use, they must be behaviourally characterised to determine their suitability in recapitulating the symptoms of the human condition. Thus, we sought to longitudinally characterise the nature, severity and time course of cognitive and behavioural changes observed in *Hdh^Q111^* heterozygous knock-in mice.To determine changes in cognition and behaviour an extensive battery of operant tests including: fixed ratio, progressive ratio, the five choice serial reaction time task and the serial implicit learning task, were applied longitudinally to *Hdh*^*Q111*^ and wild type mice. The operant test battery was conducted at 6, 12 and 18 months of age. Significant deficits were observed in *Hdh*^*Q111*^ animals in comparison to wild type animals in all operant tests indicating altered cognition (attentional and executive function) and motivation. However, the cognitive and behavioural deficits observed were not shown to be progressive over time in the longitudinal testing paradigm that was utilised. The results therefore demonstrate that the *Hdh*^*Q111*^ mouse model of HD reflects some features of the cognitive and behavioural changes shown in the human condition of HD. Although, the cognitive and behavioural deficits demonstrated were not shown to be progressive over time.

## 1. Introduction

Motor dysfunctions are core features of Huntington’s disease (HD) [1, 2], particularly in the mid and later stages of the disease. However, cognitive and behavioural changes including; lack of motivation, apathy, anxiety and reduced ability to switch tasks, have been demonstrated in HD patients, often prior to the presentation of profound motor symptoms [3–9]. Cognitive and behavioural changes such as these have been shown to significantly affect the daily activities of people living with HD, often reducing independence, causing isolation, increasing disability and reducing quality of life [10–12]. A range of knock-in mouse models of HD, have demonstrated cognitive and behavioural changes, these include specific deficits in; motivation, attention, extra dimensional set-shifting, working memory and reversal learning [13–26]. Therefore, cognitive and behavioural changes are a core early symptom of HD, that are present in many of the HD mouse lines. Thus, in order to assess whether a mouse model of HD has good face and predictive validity in terms of recapitulating the symptoms of HD, a longitudinal characterisation of the development of any associated cognitive or behavioural changes is necessary.

The *Hdh*^*Q111*^ mouse model is a knock-in line, in which the majority of exon 1 and part of intron 1 are replaced with human DNA containing ~111 CAG repeats, under the control of the endogenous murine promoter [27]. Previous studies in *Hdh*^*Q111*^ mice on a CD1 background revealed subtle differences in gait at 24 months of age [28], increased latency to fall from the rotarod at 100 weeks of age and weight loss beginning at 28 weeks of age. [29]. Anxiety like behaviour has been demonstrated in male *Hdh*^*Q111*^ mice on a CD1 background in a range of tests including; splash test, forced swim test, open field and novelty suppressed feeding at 13 weeks of age [30]. Comparatively fewer studies have been performed in *Hdh*^*Q111*^ mice on a C57 BL/6J background. Although, long term memory deficits have been observed in novel object recognition and in spatial memory in the Morris water maze task in *Hdh*^*Q111*^ mice on a C57 BL/6J background, however this study only used male animals [14]. Therefore, there is a need to longitudinally assess cognitive and behavioural change in *Hdh*^*Q111*^ mice on a C57BL/6J background strain to determine if this mouse model represents any aspects of the human condition of HD.

Cognitive and behavioural changes have previously been investigated in mouse models of HD using swimming tests including the Morris water maze [15–17, 19, 25, 31] and the water T-maze [13, 15, 31]. However, in comparison to these swimming tests, operant test batteries permit greater flexibility for testing a range of cognitive and behavioural changes in a sensitive, unbiased, rapid and automated manner. Therefore, in order to systematically and extensively explore cognition and behaviour in *Hdh*^*Q111*^ mice, a battery of operant tasks was repeated longitudinally in a group of animals which contained both wild type (WT) and heterozygous (*Hdh*^*Q111/+*^) littermate mice.

People with HD have previously been shown to be unable to acquire certain tasks [32] due to deficits in procedural learning [33], thus the ability of *Hdh*^*Q111/+*^ animals to initially learn and then subsequently re-acquire the task of ‘nose poking’ was explored using a standard fixed ratio (FR1) schedule. Furthermore, significant alterations in motivation have previously been described in other mouse models of HD [34] and in HD patients [35–37], therefore motivation was explored in *Hdh*^*Q111/+*^ animals using a progressive ratio (PR) task. In addition, different reward sizes were utilised in the PR task to explore perception of value in responding to reward and flexibility in responding to the different reward sizes. Deficits in executive functions, including spatial perception and attentional sharing have also previously been demonstrated in HD patients [4, 38–44]. Specific problems with executive function have been shown to significantly affect everyday activities for people living with HD, increasing the risk of falls and reducing quality of life [10, 12]. Thus a five-choice serial reaction time task (5-CSRTT) was included in the operant testing battery to investigate executive function in the *Hdh*^*Q111/+*^ mouse model of HD. The serial implicit learning task (SILT) (an extension of the 5-CSRTT that required an additional response to obtain a reward) was included in the test battery to probe attention, spatial awareness, and implicit learning. Previous studies in HD patients have found conflicting results with regard to whether implicit learning is affected in HD. While some have shown implicit learning impairments, prior to the onset of motor symptoms [33, 45, 46], others have demonstrated conflicting results and shown that implicit learning is unaffected in the HD patient population [47, 48].

Operant testing has previously been utilised in numerous HD mouse models to determine cognitive and behavioural dysfunction in a range of tasks, often in the absence of motor symptoms [13, 21, 24, 26, 34, 49, 50]. Although the cognitive and behavioural deficits observed in the *Hdh*^*Q111*^ mouse model of HD are yet to be longitudinally or systematically characterised. Therefore, we sought to determine the cognitive and behavioural phenotype of *Hdh*^*Q111*^ mice utilising an extensive longitudinal operant test battery which was designed to probe HD-relevant cognitive and behavioural functions.

## 2. Materials and Methods

### 2.1. Animals

All experiments were conducted in accordance with the United Kingdom Animals Scientific Procedures Act (ASPA) 1986 and subject to the local ethical review of the Animal Welfare and Ethical Review Body. From 1st January 2013, the European Union (E.U.) Directive 2010/63/EU was implemented into UK law by an update of ASPA 1986.

*Hdh*^*Q111/+*^ knock-in mice (Jax®, Bar Harbour, Maine, U.S.A.) were bred in-house on a C57BL/6J background. 16 mice, 7 *Hdh*^*Q111/+*^ (4 female and 3 male) and 9 wild type (5 female and 4 male) were used. Upon weaning, all animals were tail tipped for genotyping. A 1mm section of tail was removed using ethyl chloride anaesthetic spray (Vidant Pharma Ltd, Surrey, U.K.), samples were collected in Eppendorf tubes and shipped on dry ice to Laragen Inc. (Culver City, California, U.S.A) who performed genotyping using probe based qPCR to generate the genotype and corresponding end-point PCR to determine CAG repeat length. *Hdh*^*Q111/+*^ animals contained an average of 137 CAG repeats (ranging from 122–142 repeats). Animals were housed in mixed genotype cages with modest environmental enrichment of a single cardboard tube and a wooden chew stick. Animals were weighed and health checked weekly. Operant testing occurred between 08.00 hours and 12.00 hours, 5 days per week. 1 week prior to operant testing animals were gradually water restricted and habituated to strawberry milkshake (Yazoo® Campina Ltd, Horsham, UK) in their home cages. For the duration of operant testing animals were restricted to 3 hours of access to water per day, given after behavioural testing between 14.00 hours and 17.00 hours. Throughout the experiment animals were allowed *ad-libitum* access to laboratory chow food. A female *Hdh*^*Q111/+*^ animal became ill at approximately 13 months of age, due to health reasons unrelated to HD and was therefore humanely euthanised. At the end of the study, all animals were humanely euthanised via cervical dislocation.

### 2.2. 9-hole operant box apparatus

Operant testing was conducted in 16 9-hole operant boxes (Campden Instruments, Loughborough, UK), controlled by a BehaviourNet Controller BNC MKII operating system (Campden Instruments, Loughborough, UK), as previously described [51]. Briefly, each operant box contained a horizontal array of nine holes (11mm in diameter, placed 2mm apart and 15mm above floor level) with infrared beams localised to the front to detect nose pokes. A white light emitting device (LED) acted as the target visual stimulus at the rear of each hole. Only holes 1, 3, 5, 7 and 9 were used in testing, consequently black plastic film was used to block unused holes (2, 4, 6 and 8) and prevent their use. A peristaltic pump delivered liquid reinforcement to a magazine at the front of the box. Reward delivery to the magazine was signalled by an LED above the magazine and nose entry into the magazine was detected by an infrared beam. ‘House lights’ on the side walls of the operant chamber illuminated to signal the end of a trial or time out intervals (TOI). Background noises were provided by an extractor fan and computer operating system.

### 2.3. 9-hole operant box training

**Fig 1:**
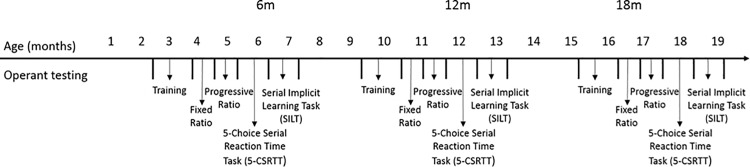
Schematic representation of operant cognitive testing timeline. Animals were tested in the operant battery at 6 month intervals at 6, 12 and 18 months of age. Animals began testing one and a half months prior to testing for that time point, due to the time it took to complete the operant test battery, animals finished testing approximately one and a half months after the required time point.

An operant test battery was conducted longitudinally, as shown in Fig 1, at 6, 12 and 18 months of age. At 10 weeks of age mice were introduced to the 9-hole operant chambers. Magazine training began with the delivery of 150μl of strawberry milk into the magazine and illumination of the magazine light, for 1 day. After successful reward retrieval, detected by an infrared beam across the magazine, the magazine light was extinguished. The process was repeated until the 20-minute session time had elapsed. After magazine training, mice were taught to nose poke on a simple fixed ratio (FR1) schedule of reinforcement. To obtain reward, mice were required to respond to a stimulus light in the central hole via a single nose poke. A correct nose poke in response to the stimulus light triggered the extinguishing of the centre light, simultaneous illumination of the magazine light and delivery of 5μl of reward into the magazine. Upon withdrawal of the animal’s nose from the magazine, the next trial was initiated. In order to promote learning the central light was painted with strawberry milk to encourage nose poking into the illuminated hole. Mice were trained on this program until they had achieved over 100 pokes into the central hole without encouragement.

### 2.4. Fixed Ratio (FR) Testing

All animals were trained on a simple FR1 schedule requiring one response for one reward. Initial, training was conducted for 13 days to allow animals to learn and acquire the nose poke response. At subsequent time points, animals were required to complete FR1 training again for 8 days to reacquire the nose poke response. After FR1 training, further FR testing was conducted, to investigate the amenability in responding to varying reward sizes of 2.5μl, 5.0μl and 7.5μl. Mice were required to poke into the central hole of the array, in response to illumination, to obtain a reward. Animals were reinforced with 5.0μl of reward for the first 3 days of testing, 2.5μl of reward for the next 3 days, before rewarding animals with 7.5μl of reward for the final 3 days of testing. The number of nose pokes into the central hole were recorded over the 45-minute session time.

### 2.5. Progressive Ratio (PR) Testing

The PR task tested the motivation of mice to obtain reward. The PR schedule of reinforcement required an increasing number of nose poke responses, into the illuminated central hole of the array to obtain successive rewards. The number of responses required to obtain reward increased by 3 after the repetition of each ratio (responses required per reward; 1, 1, 1, 3, 3, 3, 6, 6, 6, 9, 9, 9 etc). The motivation of the animals to obtain reward was recorded as the ratio attained after a 60-minute session and by measuring the response ratio attained before a defined period of non-responding (30 seconds), termed a break point.

### 2.6. 5-Choice Serial Reaction Time Task (5-CSRTT) Testing

In the 5-CSRTT animals were trained to respond, via nose poking, to a stimulus light which was randomly presented across the 5-hole array, in order to receive a 5μl reward. The task was modified from those previously described in rats [52] to be conducted in mice to investigate spatial awareness and attention. The stimulus length used became increasing shorter over the testing period, to increase the attentional load and difficulty of the task. For the first 10 days of testing a 10 second stimulus length was used, a stimulus length of 2 seconds was used for the next 5 days and a stimulus length of 0.5 seconds was used for the final 5 five days. If a response was not made within 10 seconds after the presentation of the stimulus, the light was extinguished and a time-out period of 10 seconds was initiated by illumination of the house light. The process was repeated for the 30-minute session time.

### 2.7. Serial Implicit Learning Task (SILT) Testing

For SILT testing animals were trained to respond to a 2-step sequence of lights in order to receive reward. A continuous stimulus light was randomly presented in one of the 5 holes of the array. A correct response to the first stimulus light (S1) resulted in the simultaneous extinguishing of this light and illumination of a second light (S2). A correct response to S2 resulted in the delivery of a 5μl reward into the magazine. A predictable stimulus sequence (hole 7 was always illuminated after hole 3) was embedded among other unpredictable sequences in order to probe implicit learning. The test was repeated until the 30-minute session time had elapsed.

### 2.8. Statistical Analysis

Statistical analyses were conducted in IBM SPSS Statistic 20 software. Repeated measures ANOVAs were conducted followed by simple effects analysis. Where significance was found *post-hoc* tests with Bonferroni corrections were applied to identify the locus of effects and their interaction(s). In the cases where missing values were present, missing data were estimated by an unbiased iterative interpolation procedure within the analysis. The critical significance level used throughout was α = 0.05.

## 3. Results

### 3.1. Body Weight Results

**Fig 2:**
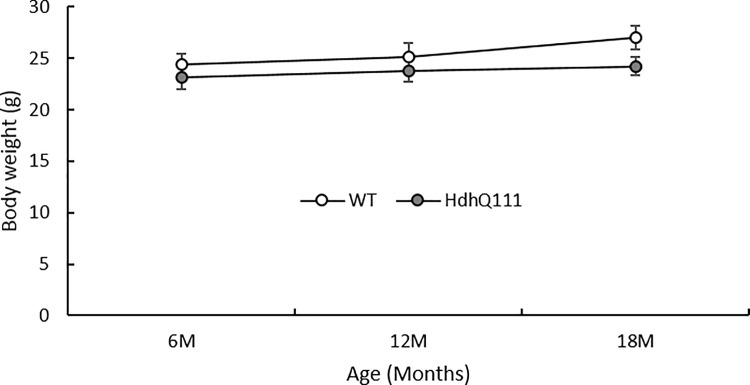
Body weight results. Body weight results revealed both wild type and *Hdh*^*Q111/+*^ animals gained body weight as they aged. Error bars represent ± standard error of the mean.

Both wild type and *Hdh*^*Q111/+*^ animals gained body weight as they aged (Fig 2: Age; F_2,26_ = 5.98, p = 0.07). Despite a trend for wild type animals to gain more weight as they aged, relative to *Hdh*^*Q111/+*^ animals, this trend failed to meet threshold levels for statistical significance (Fig 2: Genotype x Age; F_2,26_ = 3.09, p = 0.62).

### 3.2. Training Results

**Fig 3:**
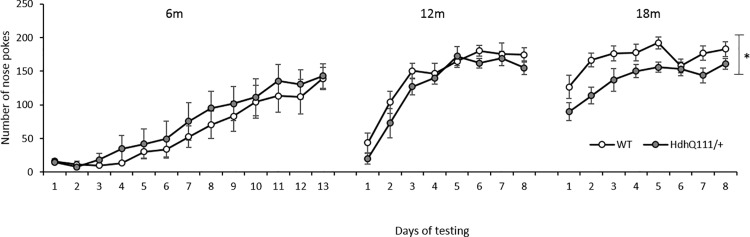
Longitudinal Operant Training Results for *Hdh*^*Q111/+*^ animals. Training at 6 months of age revealed no genotype differences but a significant main effect of time was seen. Training at 12 months of age demonstrated no significant effect of genotype, although a significant main effect of time was seen. Training at 18 months of age revealed *Hdh*^*Q111/+*^ animals made significantly fewer nose poke responses than wild type animals. An overall significant effect of time was seen. Data are shown for a total of 16 mice, 7 *Hdh*^*Q111+*^ and 9 wild type. Data are presented as the average number of nose poke responses made by either *Hdh*^*Q111/+*^ or wild type mice across each day of testing at each time point. Statistical analyses were performed separately for each testing time point. Error bars represent ± standard error of the mean, * p<0.05.

During initial training in the nose poke response, at the first 6-month time point, no significant differences in performance were demonstrated between wild type and *Hdh*^*Q111/+*^ animals (Fig 3: Genotype; F_1,12_ = 0.51, p = n.s.). However, all animals made an increasing number of nose poke responses over time and subsequent training days (Fig 3: Time; F_12_,_144_ = 24.15, p<0.001), as they successfully acquired and learnt the nose poke response.

Animals were re-trained for 8 days at 12 months of age for the second testing time point (Fig 3). *Hdh*^*Q111/+*^ animals were able to reacquire the nose poke response to the same degree as wild type animals (Fig 3: Genotype; F_1,12_ = 3.01, p = n.s.), and as was seen at the earlier time point, a significant overall effect of time was demonstrated (Fig 3: Time; F_7,84_ = 44.67, p<0.001), with the overall number of responses increasing over subsequent testing days.

At the final 18-month time point, animals were placed on the training schedule for 8 days (Fig 3). *Hdh*^*Q111/+*^ animals were unable to make as many nose poke responses as wild type animals over the 8 days of training (Fig 3: Genotype; F_1,12_ = 5.85, p<0.05). Thus indicating that *Hdh*^*Q111/+*^ animals were now unable to reacquire the nose poke response in comparison to wild type animals. A significant overall effect of time was seen, as animals learnt to reacquire the nose poke response on subsequent days of training (Fig 3: Time; F_7,48_ = 4.217, p<0.05).

### 3.3. Fixed Ratio (FR) Results

**Fig 4:**
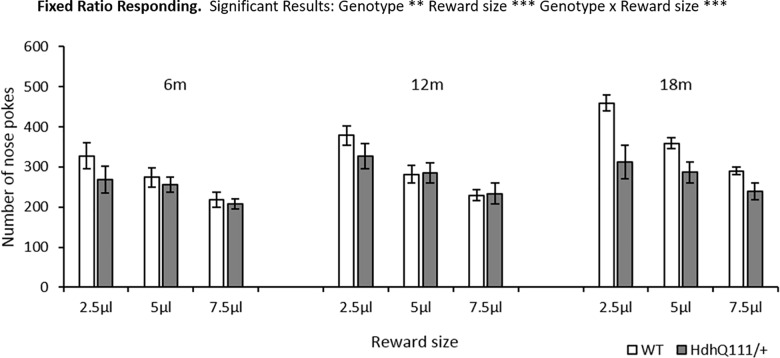
Longitudinal Fixed Ratio (FR) Results for *Hdh*^*Q111/+*^ animals. FR results demonstrated a significant overall effect of reward size, with animals responding more for smaller reward sizes. *Hdh*^*Q111/+*^ animals made significantly fewer responses than wild type animals when all testing conditions were considered. *Hdh*^*Q111/+*^ animals made significantly fewer responses than wild type animals, which was driven by the result at the 18-month time point. Data are shown for a total of 16 mice, 7 *Hdh*^*Q111+*^ and 9 wild type animals. Data are shown as the average number of responses made over 3 days of testing. Error bars represent ± standard error of the mean. Significance considers genotypic differences when all ages of testing and reward sizes are considered. ** p<0.01, *** p<0.001.

Once all animals had successfully learnt to nose-poke or had reacquired the poking response, the testing program was manipulated to investigate the effects of using different reward sizes on responding. Animals made significantly more responses at the smaller reward sizes (Fig 4: Reward size; F_2,24_ = 105.64, p<0.001). When all testing ages and reward sizes were considered. *Hdh*^*Q111/+*^ animals made significantly less responses than wild type animals (Fig 4: Genotype; F_1,12_ = 11.355, p<0.01) and despite a trend for *Hdh*^*Q111/+*^ animals to make less responses than wild type animals over time, no significant interaction effect was demonstrated (Fig 4: Age x Genotype; F_2,24_ = 3.078, p = 0.065). *Hdh*^*Q111/+*^ animals made significantly fewer responses to the smallest 2.5μl reward in comparison to wild type animals when all time points were considered (Fig 4: Genotype x Reward size; F_2,24_ = 11.88, p<0.001), this effect was largely driven by the results seen at the 18-month time point.

*Hdh*^*Q111/+*^ animals made a similar number of responses as wild type animals at each reward size at the 6-month time point and 12-month time point, as shown in Fig 4. However, at the 18-month time point, *Hdh*^*Q111/+*^ animals made less responses than wild type animals at all reward sizes.

### 3.4. Progressive Ratio (PR) Results

**Fig 5:**
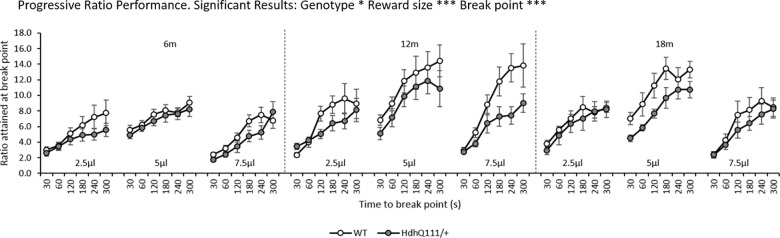
Longitudinal Progressive Ratio (PR) Results for *Hdh*^*Q111/+*^ animals. PR testing of motivation demonstrated no statistically significant differences in performance between *Hdh*^*Q111/+*^ and wild type animals at any age or reward size considered. However, animals were able to reach higher ratios of attainment at larger reward sizes and at higher break points. Data are shown for a total of 16 mice, 7 *Hdh*^*Q111+*^ and 9 wild type. Data are shown as the average number of responses made over 3 days of testing, for each reward size. Error bars represent ± standard error of the mean. * p<0.05, ** p<0.01, *** p<0.001.

Animals were able to reach a higher ratio of responding in PR testing when larger reward sizes were used in testing (Fig 5: Reward size; F_2,24_ = 46.53, p<0.001) and animals were able to reach higher ratios of responding at higher break points (Fig 4: Break point; F_5,60_ = 116.99, p<0.001), as seen in Fig 5. *Hdh*^*Q111/+*^ animals demonstrated a reduced ratio of attainment in comparison to wild type animals (Fig 5: Genotype; F_1,12_ = 4.26, p<0.05), although this deficit was not shown to be progressive over time (Fig 5: Age x Genotype; F_2,24_ = 0.542, p = n.s.).

### 3.5. Five Choice Serial Reaction Time Task (5-CSRTT) Results

**Fig 6:**
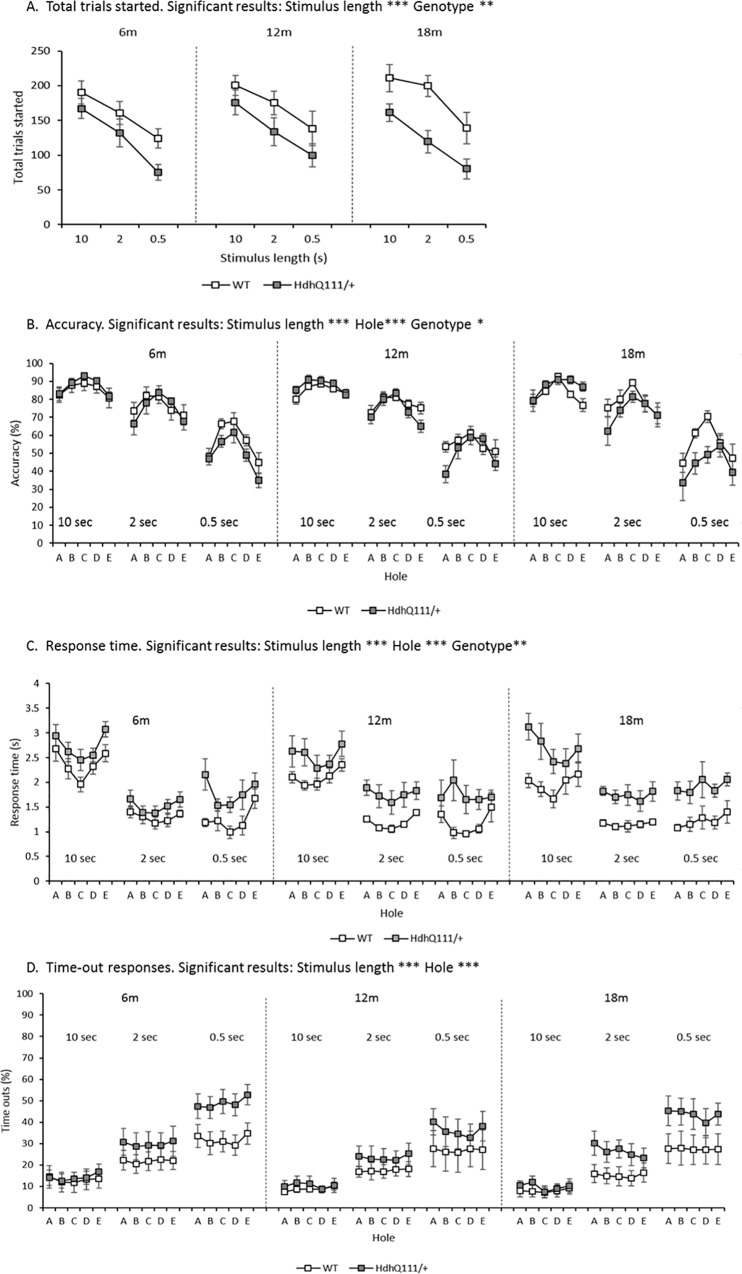
5-Choice Serial Reaction Time Task (5-CSRTT) Results Longitudinally for *Hdh*^*Q111/+*^ animals over 18 months of testing. A. Total trials started. Animals initiated significantly fewer trials as the stimulus length was decreased, at all ages. *Hdh*^*Q111/+*^ animals initiated significantly fewer trials than wild type animals, largely due to the magnitude of difference in the number of initiated trials at 18 months of age. B. Accuracy. Animals were significantly less accurate in responding at shorter stimulus lengths and were significantly more accurate is responding to the central hole in comparison to the peripheral holes. *Hdh*^*Q111/+*^ animals were significantly less accurate than wild type animals, although no genotype by age interaction was demonstrated. C. Response time. Animals responded faster to the shorter stimulus lengths and into the central hole in comparison to peripheral holes. *Hdh*^*Q111/+*^ animals were significantly slower to respond to the stimulus than wild type animals. D. Time out responses. Animals made significantly more time outs as stimulus length decreased. Animals also made significantly more time outs at the peripheral holes in comparison to the central hole. Data are shown for a total of 16 mice, 7 *Hdh*^*Q111/+*^ and 9 wild type and is the average number of responses made over 5 days of testing at each stimulus length. Error bars represent ± standard error of the mean. * p<0.05, ** p<0.01, *** p<0.001.

Animals initiated significantly fewer trials in the 5-CSRTT when the stimulus length was decreased and all testing ages were considered (Fig 6A: Stimulus length; F_2,24_ = 118.49, p<0.001). *Hdh*^*Q111/+*^ animals initiated significantly fewer trials than wild type animals when all ages and stimulus lengths were considered (Fig 6A: Genotype; F_1,12_ = 10.14, p<0.01), although no significant interaction with age was demonstrated (Fig 6A: Age x Genotype; F_2,24_ = 0.43, p = n.s.). Decreasing the stimulus length led animals to make significantly fewer accurate responses (Fig 6B: Stimulus length; F_2,24_ = 291.86, p<0.001). Furthermore, animals were significantly more accurate in responding into the central hole in comparison to the peripheral holes in all testing conditions (Fig 6B: Hole; F_4,48_ = 25.12, p<0.001). When all testing ages were included in the analysis, *Hdh*^*Q111/+*^ animals were significantly less accurate in responding in the 5-CSRTT in comparison to wild type animals (Fig 6B: Genotype; F_1,12_ = 5.00, p<0.05) and this deficit was stable over time (Fig 6B: Age x Genotype; F_2,24_ = 0.33, p = n.s.). However, the overall genotype difference was largely driven by deficits observed at the shortest 0.5 second stimulus length, rather than at the longer stimulus lengths of 10 seconds and 2 seconds (Fig 6B).

Animals were significantly faster to respond in the 5-CSRTT when the stimulus length was decreased (Fig 6C: Stimulus length; F_2,24_ = 108.18, p<0.001). This effect was due to an increase in response time at the 10 second stimulus length, in comparison to the response time at the 2 second and 0.5 second stimulus lengths, at all ages of testing (Fig 6C). Furthermore, animals were significantly faster to respond into the central hole of the array in comparison to the peripheral holes in all testing conditions (Fig 6C: Hole; F_4,48_ = 12.01, p<0.001).

*Hdh*^*Q111/+*^ animals were significantly slower to respond to stimulus in the 5-CSRTT than wild type animals (Fig 6C: Genotype; F_1,12_ = 13.19, p<0.01), however no statistically significant interaction effect was observed (Fig 6C: Age x Genotype; F_2,24_ = 0.23, p = n.s.).

In the 5-CSRTT when the stimulus length was decreased, the number of time out responses made by animals significantly increased (Fig 6D: Stimulus length; F_2,24_ = 82.54, p<0.001). Furthermore, animals made more time out responses when responding into the peripheral holes in comparison to responses made into the central hole (Fig 6D: Hole; F_4,48_ = 6.82, p<0.001). The number of time out responses made did not significantly differ between *Hdh*^*Q111/+*^ and wild type animals (Fig 6D: Genotype; F_1,12_ = 3.81, p = n.s.).

### 3.6. Serial Implicit Learning Task (SILT) Results

**Fig 7:**
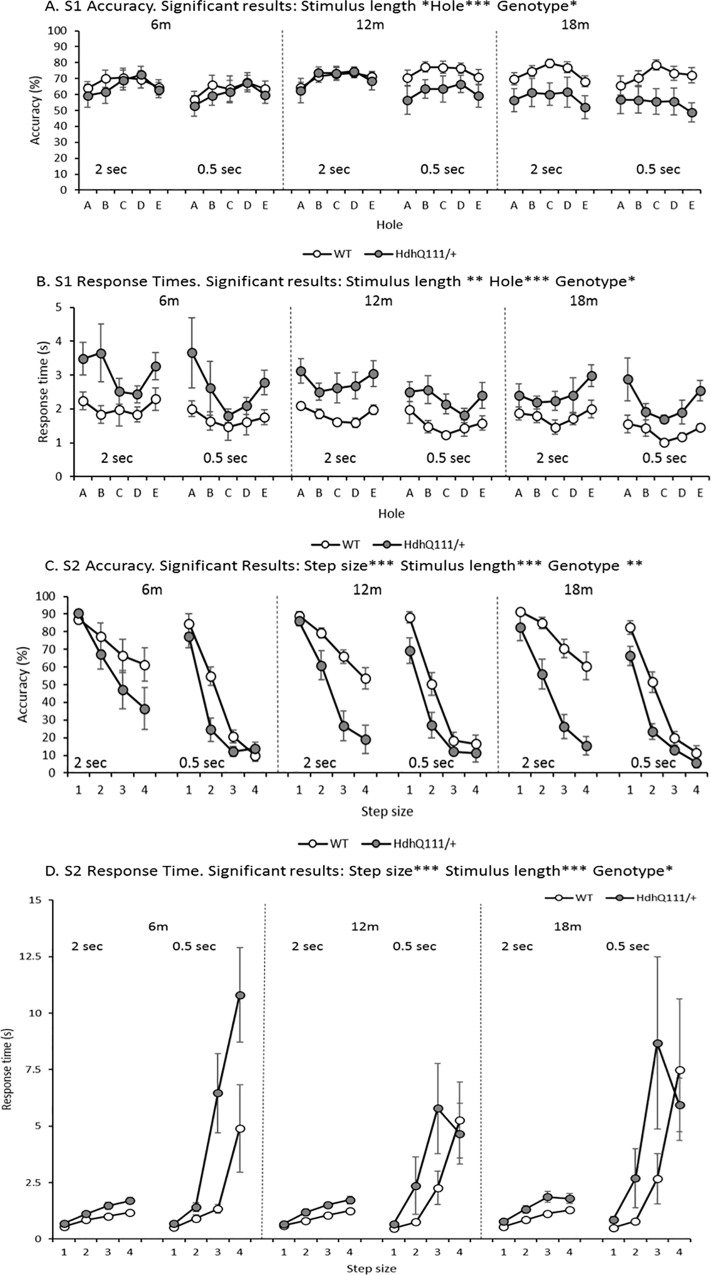
Serial Implicit Learning Task (SILT) results for accuracy and response time in *Hdh*^*Q111/+*^ animals longitudinally. A. S1 Accuracy. Animals were more accurate in responding into the central hole at shortest (2 second) stimulus length. B. S1 Response Time. Animals were faster to respond into the central hole at the shorter 0.5 second stimulus length. *Hdh*^*Q111/+*^ animals were significantly slower to respond across all task manipulations in comparison to wild type animals. C. S2 Accuracy. *Hdh*^*Q111/+*^ animals were significantly less accurate in comparison to wild type animals, although there was no interaction with time. D. S2 Response Time. Animals took significantly longer to respond at the shorter 0.5 second stimulus length and as the step size increased. *Hdh*^*Q111/+*^ animals were significantly slower to respond than wild type animals when all testing conditions were considered. Data are shown for a total of 16 mice, 7 *Hdh*^*Q111/+*^ and 9 wild type and is the average number of responses made over 5 days of testing at each stimulus length. Error bars represent ± standard error of the mean. * p<0.05, ** p<0.01, *** p<0.001.

SILT analysis comprised of S1 and S2 accuracy and response time measures to probe attention and motor function, as well as accuracy and response time measures for the predictable sequence as a measure of implicit learning. Animals were significantly less accurate in S1 responding accuracy in the SILT when the stimulus length was decreased from 2 seconds to 0.5 seconds (Fig 7A: Stimulus length; F_1,12_ = 6.53, p<0.05). Furthermore, animals were significantly more accurate in responding into the central hole rather than the peripheral holes (Fig 7A: Hole; F_4,48_ = 19.27, p<0.001). *Hdh*^*Q111/+*^ animals were significantly slower in their response times than wild type animals (Fig 7A: Genotype; F_1,12_ = 8.40, p <0.05). However, this deficit was stable over time (Fig 7A: Age x Genotype; F_2,24_ = 2.66, p = n.s.).

Animals were significantly faster to respond to the S1 stimulus when the stimulus length was reduced (Fig 7B: Stimulus length; F_1,12_ = 11.70, p<0.01) at all ages. Furthermore, animals were significantly faster to respond into the central hole rather than the peripheral holes (Fig 7B: Hole; F_4,48_ = 11.60, p<0.001). When all testing ages were considered, *Hdh*^*Q111/+*^ animals showed an increased S1 response time in comparison to wild type animals (Fig 7B: Genotype; F_1,12_ = 8.41, p<0.05), but this deficit was not shown to be progressive over time (Fig 7B: Genotype x Age; F_2,24_ = 0.160, p = n.s.). In the second response required in the SILT task (S2), mice were significantly less accurate to respond when the step size of the response was increased (Fig 7C: Step; F_3,36_ = 228.22, p<0.001) and when the stimulus length was decreased (Fig 7C: Stimulus length; F_1,12_ = 169.19, p<0.001). *Hdh*^*Q111/+*^ animals were significantly less accurate in S2 responding in comparison to wild type animals when all ages were considered (Fig 7C: Genotype; F_1,12_ = 14.76, p<0.01), although this deficit was not shown to be progressive over time (Fig 7C: Age x Genotype; F_2,24_ = 4.31, p = n.s.).

Animals were significantly slower to respond to the S2 stimulus when the step size was increased (Fig 7D: Step; F_3,36_ = 42.35, p<0.001) and when the stimulus length was decreased (Fig 7D: Stimulus length; F_1,12_ = 33.44, p<0.001). *Hdh*^*Q111/+*^ animals were shown to be significantly slower to respond to the S2 stimulus when all ages were considered (Fig 7D: Genotype; F_1,12_ = 6.45, p<0.05), this finding was driven by the results obtained using the 2 second stimulus length and was consistent over time (Fig 7D: Age x Genotype; F_2,24_ = 0.43, p = n.s.).

**Fig 8:**
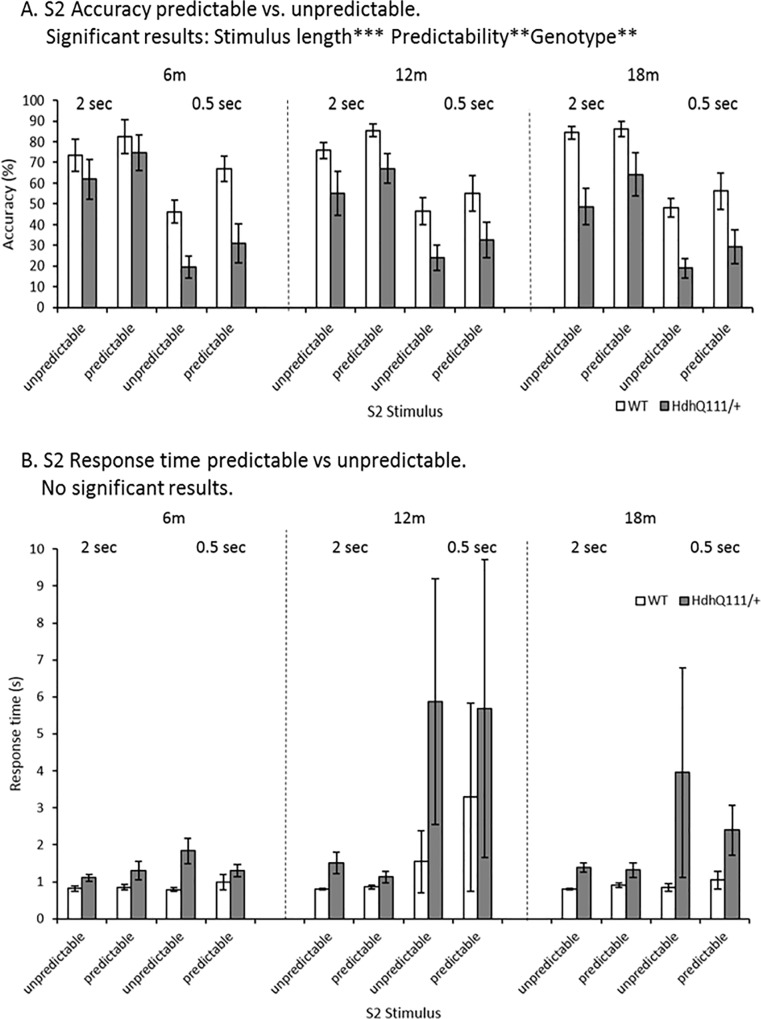
Serial Reaction Time Task (SILT) Results for Accuracy and Response Time for predictability of stimulus in *Hdh*^*Q111/+*^ animals longitudinally. A. S2 Accuracy. Mice were significantly more accurate when the duration of the stimulus length was increased and when the stimulus was predictable rather than unpredictable *Hdh*^*Q111/+*^ animals were significantly less accurate overall in responding in comparison to wild type animals The predictability of the stimulus did not confer any significant benefit to either wild type animals or *Hdh*^*Q111/+*^ animals. B. S2 Response Time. The predictability of the S2 stimulus in the SILT did not confer any significant advantage to how rapidly animals were able to respond. *Hdh*^*Q111/+*^ animals showed a trend to be significantly slower than wild type animals in all testing conditions, this trend failed to meet the threshold for statistical significance. Data are shown for a total of 16 mice, 7 *Hdh*^*Q111/+*^ and 9 wild type and is the average number of responses made over the final 5 days of testing at each stimulus length. Error bars represent ± standard error of the mean. * p<0.05, ** p<0.01, *** p<0.001.

S2 accuracy in responding to the stimulus was considered in light of the predictability of the stimulus, which was designed to probe implicit learning (Fig 8A). Mice were significantly less accurate when the stimulus length was decreased (Fig 8A: Stimulus length; F_1,12_ = 247.91, p<0.001). Furthermore, animals were significantly more accurate when the stimulus was predictable rather than unpredictable (Fig 8A: Predictability; F_1,12_ = 17.04, p<0.01). *Hdh*^*Q111/+*^ animals were significantly less accurate overall in responding in comparison to wild type animals regardless of the predictability of the stimulus (Fig 8A: Genotype; F_1,12_ = 12.61, p<0.01). However, the predictability of the stimulus did not confer any significant benefit to either wild type animals or *Hdh*^*Q111/+*^ animals (Fig 8A: Genotype x Predictability; F_1,12_ = 0.45, p = n.s.).

The predictability of the S2 stimulus in the SILT did not confer any significant advantage to how rapidly animals were able to respond (Fig 8B: Predictability; F_1,12_ = 0.27, p = n.s.). There was considerable variation among response times, in some instances, therefore no significant effect of stimulus length was seen (Fig 8B: Stimulus length; F_1,12_ = 0.15, p = n.s.). Although *Hdh*^*Q111/+*^ animals showed a trend to be significantly slower than wild type animals in all testing conditions, this trend failed to meet the threshold for statistical significance (Fig 8B: Genotype; F_1,12_ = 4.328, p = 0.062).

## 4. Discussion

Longitudinal operant testing of *Hdh*^*Q111/+*^ animals revealed cognitive and behavioural deficits in comparison to wild type animals in fixed ratio (FR), progressive ratio (PR), 5-choice serial reaction time task (5-CSRTT) and serial implicit learning task (SILT), although these deficits were not demonstrated to be progressive over time. The results of this longitudinal operant test battery therefore suggest that the *Hdh*^*Q111/+*^ mouse demonstrates an insidious and subtle disease progression; as cognitive and behavioural deficits were not seen in the early stages of the disease as in the human condition (3–9). However, this may be explained by the nature of the longitudinal study design, as training *Hdh*^*Q111/+*^ animals on operant tasks at a young age has been shown to modify subsequent HD related cognitive symptoms [51]. Despite this, the nature of longitudinal testing requires repeated operant testing at multiple time points to appropriately assess the progression of cognitive and behavioural symptoms over time. Furthermore, the relatively small sample size used in this study needs to be carefully considered in the interpretation of the results.

No statistically significant differences in body weight were observed between *Hdh*^*Q111/+*^ mice, relative to wild type mice. Although, in a previous study [51] progressive weight loss was detected in *Hdh*^*Q111/+*^ mice, relative to wild type mice, from 11 months of age. However, the previous longitudinal study of *Hdh*^*Q111/+*^ animals, included a much larger experimental cohort. Therefore, the comparatively small number of animals used in the present study is important to consider in the interpretation of the results.

Initial training and reacquisition of the nose poke response demonstrated an inability for *Hdh*^*Q111/+*^ animals to initiate as many trials as wild type animals at the oldest 18-month time point which may indicate the onset of motor abnormalities. This observation is also reflected in the results of the 5-CSRTT, with *Hdh*^*Q111/+*^ animals initiating significantly less trials than wild type animals. Motor deficits have been previously demonstrated in *Hdh*^*Q111/+*^ mice [53] from 9 months of age and thus the reduction in responding may be due to significant motor impairments which are also reflected in the response time measures. Therefore, *Hdh*^*Q111/+*^ animals may not be physically able to initiate a response as rapidly as wild type animals. It may also be the case that the response time deficits observed in *Hdh*^*Q111/+*^ animals are reflective of an inability to initiate movement. Difficulties in the initiation of movement have been described in *Hdh*^*Q111/+*^ animals progressively from 9 months of age on the balance beam [53]. Although the initiation of movement has not yet been thoroughly explored in HD mouse models, people with HD have showed a decrease in the initiation of movement from early in the disease progression [54, 55].

Upon the introduction of different reward sizes into the FR1 testing schedule a general effect of reward size was seen, with all animals making significantly more responses for smaller reward sizes. This effect may be due to animals taking a greater time to consume larger rewards, or there may be an effect of satiation, with animals becoming satiated when they consume larger rewards. However, for the 2.5μl reward size *Hdh*^*Q111/+*^ animals responded less than wild type mice which was largely due to differences observed at 18 months of age. This may be due to an underlying apathetic phenotype in *Hdh*^*Q111/+*^ animals, which means that they respond less than wild type animals for smaller rewards. However, this may also be explained by deficits in motivation, as demonstrated in the progressive ratio task, with *Hdh*^*Q111/+*^ animals displaying decreased motivation to obtain reward in comparison to wild type animals. These results are consistent with those previously observed in the Hdh^Q92^ [56], zQ175 and BAC mouse models [34] which demonstrated reduced motivation in HD mice in comparison to wild type controls at 15 months and 73 weeks respectively. However, there are some differences to the previous studies, which are important to consider. Food restriction was used rather than water restriction and different versions of the PR task were used, which required higher ratios of responding to obtain reward. Furthermore, previous studies often conduct the PR task in animals at a single time point and thus it may be the case that if *Hdh*^*Q111/+*^ animals were tested solely at 18 months of age, rather than longitudinally, they would have a greater motivational deficit in the PR task in comparison to wild type animals. The motivational deficits observed are consistent with previous studies in the human patient population [35–37], although these studies often demonstrate motivational problems early in the disease progression of HD, prior to the onset of motor symptoms, which progress over time.

In the 5-CSRTT, the inability of *Hdh*^*Q111/+*^ animals to initiate as many trials as wild type animals is indicative of a general inability to complete the task. The precise reason for this may be due to multiple underlying behavioural or psychological factors, such as depression or apathy. But, given the considerable motor dysfunction that has been demonstrated in *Hdh*^*Q111/+*^ animals at 18 months of age [53], it is perhaps the case that animals are simply unable to physically get to the stimulus to make a response within the required time.

*Hdh*^*Q111/+*^ animals were able to maintain their response accuracy in the 5-CSRTT over consecutive testing ages, despite considerable response time deficits. Therefore, *Hdh*^*Q111/+*^ animals are able to maintain their accuracy in responding in the 5-CSRTT, at the detriment of responding slower to the stimulus. This suggestion is also supported the fact that *Hdh*^*Q111/+*^ animals did not demonstrate a significant difference in the number of time out responses made in comparison to wild type animals. Therefore, *Hdh*^*Q111/+*^ animals are able to respond to the stimulus and to the same degree of accuracy as wild type animals, although it takes them significantly longer to do so.

In the SILT *Hdh*^*Q111/+*^ animals were significantly slower in responding to both the S1 and S2 stimuli, than wild type animals, thus further reflecting motor deficits previously observed in *Hdh*^*Q111/+*^ animals [53]. The S1 accuracy deficits demonstrated in *Hdh*^*Q111/+*^ animals at 18 months of age were surprising, given that these deficits did not exist in the 5-CSRTT. Therefore, it may be the case that when the complexity of the operant task is increased and requires two responses, animals are unable to maintain the accuracy of responding, as was shown in the 5-CSRTT. It could also be that motor dysfunction prevents animals from responding to the S1 SILT stimulus accurately. Although, the deficits observed in the SILT task in S2 accuracy among *Hdh*^*Q111/+*^ animals may be attributed to motor problems, these deficits may further be explained by the eccentricity of response required in this task, which has been previously demonstrated in rats with striatal lesions [57].

In the SILT task animals were unable to obtain any benefit from the implicit learning sequence within the task in terms of S2 accuracy or S2 response time. Therefore, there was an inability for animals to learn the implicit component of the task. Previous attempts to explore implicit learning deficits in HD patients have demonstrated mixed results, while some have shown clear implicit learning impairments, prior to the onset of motor symptoms [33, 45, 46], others have concluded that implicit learning is unaffected in the HD patient population [47, 48]. However, as animals were unable to learn the implicit component of the SILT task we cannot conclude if the *Hdh*^*Q111/+*^ mouse demonstrated any impairments in implicit learning.

The experimental design utilised in the present study, ensured that exposure to the operant boxes was the same among animals. Although, *Hdh*^*Q111/+*^ animals began significantly fewer trials than wild type animals, which meant that wild type animals were exposed to comparatively more reward and thus practice effects may have been demonstrated within the data. Therefore, in future longitudinal experiments that test cognitive and behavioural change, it may be necessary to design the operant tasks so that each animal is required to complete a set number of trials, rather than a particular session time, during the task to eliminate any possible practice effects.

In addition, the longitudinal operant testing battery was performed such that the same animals underwent repeated testing in the operant boxes. Therefore, animals were extensively exposed to the operant boxes, responding, obtaining of reward and the associated water restriction for an extended period of time. As previously described in *Hdh*^*Q111/+*^ mice, testing animals on operant tasks from a young age can significantly modify subsequent HD related behaviours [51] which may have inadvertently prevented some cognitive decline. Thus this may explain why the cognitive and behavioural deficits observed were not progressive over time. However, in order to study cognition and behaviour longitudinally repeated testing is required. In addition, a comparatively small number of animals were used in this study, thus, in future studies, it would be beneficial to test larger cohorts of animals to increase the robustness of the results that are obtained.

In summary, the extensive longitudinal cognitive and behavioural assessments presented here, demonstrate cognitive and behavioural deficits in *Hdh*^*Q111/+*^ animals in comparison to wild type animals in operant tasks of motivation, attention and executive function. However, the cognitive and behavioural deficits observed were not progressive over time. These findings suggest that the *Hdh*^*Q111/+*^ mouse model of HD demonstrates a cognitive and behavioural profile that models some aspects of HD, but does not represent the progressive nature of the cognitive and behavioural decline demonstrated in the human condition.

## Supporting Information

S1 File Data Sets for Operant Testing. Complete data sets for longitudinal operant testing including Fixed Ratio (FR), Progressive Ratio (PR), 5-Choice Serial Reaction Time Task (5-CSRTT) and Serial Implicit Learning Task (SILT).
(XLSX)
